# Tafenoquine for the radical cure and prevention of malaria: the importance of testing for G6PD deficiency

**DOI:** 10.5694/mja2.50474

**Published:** 2020-02-09

**Authors:** Robert J Commons, James S McCarthy, Ric N Price

**Affiliations:** ^1^ Menzies School of Health Research Darwin NT; ^2^ WorldWide Antimalarial Resistance Network Darwin NT; ^3^ Ballarat Health Services Ballarat VIC; ^4^ QIMR Berghofer Medical Research Institute Brisbane QLD; ^5^ University of Queensland Brisbane QLD; ^6^ Centre for Tropical Medicine and Global Health Nuffield Department of Clinical Medicine University of Oxford Oxford UK; ^7^ Mahidol University Bangkok Thailand

**Keywords:** Malaria, Anti‐infective agents

Qualitative tests alone are not sufficient for safe prescribing of tafenoquine

The cure of patients with *Plasmodium vivax* malaria requires killing both the asexual stages of the parasites in the blood as well as the dormant liver stages (hypnozoites) — together known as radical cure. Until recently, primaquine was the only available hypnozoiticidal agent. However, in 2018, the Therapeutic Goods Administration in Australia and the Food and Drug Administration in the United States granted licences for tafenoquine for the radical cure of *P. vivax* malaria and for malaria chemoprophylaxis. Both primaquine and tafenoquine are 8‐aminoquinoline antimalarial drugs that can cause severe haemolysis in individuals with glucose‐6‐phosphate dehydrogenase (G6PD) deficiency.^1^, ^2^ Primaquine is rapidly metabolised and eliminated from the body, with a half‐life of 6 hours.^2^ Radical cure of latent infection is achieved with daily administration, the total dose spread over 14 days to improve tolerability. In contrast, tafenoquine has a long elimination half‐life (14 days)^3^ and can be prescribed as a single‐dose for radical cure of *P. vivax*, or as a convenient weekly regimen for the chemoprophylaxis of malaria.^4^

Tafenoquine has been approved for both radical cure and antimalarial prophylaxis, although each indication has a different formulation.^5^ For prophylaxis, tafenoquine is available in a 100 mg tablet (Kodatef [60 Degrees Pharmaceuticals]), and administered as a 3‐day loading dose followed by weekly dosing thereafter (Box 1). For the radical cure of *P. vivax*, tafenoquine is available in a 150 mg tablet (Kozenis [GlaxoSmithKline]) as a single dose regimen (Box 1). The long elimination half‐life of tafenoquine enables a pragmatic single dose regimen for radical cure, but it has potential to cause prolonged and severe haemolysis in vulnerable individuals.^6^

Box 1 **Recommended dosing for tafenoquine**

**Table:** 

Dosing for the chemoprophylaxis of malaria: Loading dose: 200 mg daily for 3 days before entering the malaria endemic area Maintenance dose: 200 mg weekly starting 7 days after the last loading dose Final dose: Final 200 mg weekly dose to be given in the week after leaving the malaria endemic area
Dosing for the radical cure of *Plasmodium vivax*: Single 300 mg dose in conjunction with blood schizontocidal treatment (eg, artemether–lumefantrine)

G6PD deficiency is an X‐linked genetic disorder and presents at a frequency of up to 30% in some populations. Over 180 different genotypes have been associated with varying degrees of enzyme deficiency.^7^, ^8^ While hemizygous males and homozygous deficient females have activity below 30%, heterozygous females have intermediate activity between 30% and 70%, the extent of which is determined by lyonisation in embryonic development.^8^ Lyonisation is the random cellular inactivation of one of the copies of the X chromosome, leading to variation in the number of transcriptionally active genes in the progeny of each of the embryonal cells.

National and international guidelines recommend that all patients should be assessed for G6PD deficiency before prescription of primaquine or tafenoquine.^9^, ^10^ The diagnosis of G6PD deficiency can be made either using a test that provides a categorical assessment of deficiency (qualitative tests) or one that provides a continuous measure of enzyme activity (quantitative tests). The gold standard for the diagnosis of G6PD deficiency is a quantitative assay using spectrophotometry, although novel point‐of‐care devices for quantitative assessment have recently been validated and marketed.^11^, ^12^ Qualitative tests include the fluorescent spot test and point‐of‐care rapid diagnostic tests (RDTs) using lateral flow technology, such as the CareStart G6PD RDT (Access Bio). While the qualitative tests require less laboratory resources and can diagnose severe G6PD deficiency (< 30% activity), they are unable to reliably identify female heterozygotes with deficiency in the intermediate range.^13^

Primaquine can be prescribed to patients who are not severely G6PD‐deficient (ie, those with ≥ 30% enzymatic activity), and can therefore be prescribed on the basis of a qualitative test result.^10^ Individuals with enzymatic activity below 30% should not be prescribed the 14‐day primaquine regimen. Since clinically relevant haemolysis can still occur in patients with intermediate G6PD deficiency (30% to < 70% activity),^8^ prescribers should be aware of the risk of haemolysis in individuals with intermediate deficiency, and if this occurs, primaquine should be terminated early. Since tafenoquine is eliminated slowly, it can cause more prolonged exposure to oxidative stress that cannot be mitigated by drug cessation. Therefore, the recommended threshold of enzyme activity for tafenoquine prescription is more stringent, and females with intermediate G6PD activity (< 70% enzymatic activity) should be excluded based on the pivotal clinical studies performed for the licensing of tafenoquine for radical cure.^14^, ^15^, ^16^

The approach to diagnosing G6PD deficiency varies slightly between diagnostic laboratories and this may cause confusion to prescribers regarding a patient's G6PD status. Of almost 140 diagnostic laboratories in Australia that offer screening for G6PD deficiency, the majority use a qualitative test first and usually only send samples for confirmation with a quantitative test if the qualitative test indicates deficiency. Laboratories using quantitative spectrophotometric assays currently report G6PD activity in international units per gram of haemoglobin, providing a reference range rather than presenting the percentage activity. Although the reference range for these quantitative tests varies with specific assays and laboratories, the lower limit of the normal range typically lies close to the upper threshold for intermediate G6PD activity (60–80%). Hence, tafenoquine can be prescribed safely to an individual with a quantitative result that demonstrates G6PD activity within the reference range (G6PD normal). While quantitative testing may not be available on‐site at all clinical laboratories, in general, the initiation of an 8‐aminoquinoline antimalarial drug is not urgent, since acute management of *P. vivax* malaria is achieved with a schizontocidal drug and radical cure can be prescribed later when the results of the quantitative assay are available. In malaria endemic regions, the requirement for a quantitative test currently prevents widespread diagnosis of G6PD deficiency at the more conservative threshold required for prescribing tafenoquine. It is hoped that the recent availability of novel quantitative point of care tests will facilitate greater access to the drug. Clinicians also need to be aware that tafenoquine is contraindicated, or not yet recommended, in children (Kozenis is licensed for radical cure in patients aged ≥ 16 years and Kodatef is licensed for chemoprophylaxis in patients aged ≥ 18 years), and in pregnant and breastfeeding women.

Tafenoquine has been shown to be non‐inferior to low dose primaquine regimens (15 mg daily or about 0.25 mg/kg/day for 14 days) for radical cure of *P. vivax* in multicentre randomised controlled trials.^15^, ^16^ It should be noted that these trials did not account for reduced adherence and effectiveness of a prolonged primaquine regimen under programmatic conditions and did not compare tafenoquine with high dose primaquine regimens (eg, 0.5 mg/kg/day for 14 days), which are recommended in East Asia and Oceania.^10^ Safety profiles for tafenoquine and primaquine were similar in these studies, when treatment was restricted to patients with normal G6PD activity (≥ 70%).^15^, ^16^

In summary, the registration of tafenoquine represents an important advance for radical cure of malaria due to its convenient dosing, and adds an additional weekly option for malaria chemoprophylaxis. However, before prescribing the drug, clinicians need to request a quantitative G6PD test to exclude patients with either severe or intermediate enzyme activity (Box 2). New point‐of‐care tests are now available and likely to be marketed within Australia in the near future, allowing the rapid identification of individuals with intermediate or severe G6PD deficiency. In the meantime, laboratories should state the methodology by which G6PD deficiency was assessed and refer to a reference laboratory for definitive diagnosis if quantitative testing is unavailable locally.

Box 2 **Options for radical cure of *Plasmodium vivax* according to glucose‐6‐phosphate dehydrogenase (G6PD) activity**

* Compared with the adjusted male median G6PD activity of the population. ^†^ Weekly primaquine may not be appropriate in all individuals with G6PD activity < 30%. In these patients, specialist advice should be sought or expectant observation may be undertaken, with subsequent treatment of clinical relapses.

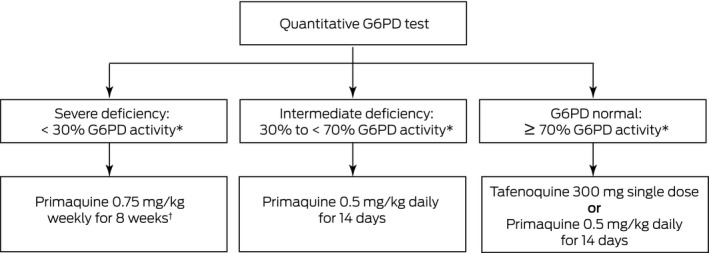

## Acknowledgements

Ric Price is a Wellcome Trust Senior Fellow in Clinical Science (200909).

## Competing interests

Ric Price was on the expert panel for the tafenoquine licensing application at the US Federal Drug Administration and on the expert committee for the Australian Therapeutic Goods Administration. His travel expenses to the US were reimbursed by GlaxoSmithKline. James McCarthy received funding from 60 Degrees Pharmaceuticals to conduct a randomised controlled trial to test the activity of tafenoquine as a potential drug for the chemoprophylaxis of malaria.

## Provenance

Not commissioned; externally peer reviewed.

## References

[1] Baird JK . Primaquine toxicity forestalls effective therapeutic management of the endemic malarias. Int J Parasitol 2012; 42: 1049–1054. 22968164 10.1016/j.ijpara.2012.06.006

[2] Baird JK . Tafenoquine for travelers’ malaria: evidence, rationale and recommendations. J Travel Med 2018; 25: tay100. 10.1093/jtm/tay110 PMC6243017 30380095

[3] Brueckner RP , Lasseter KC , Lin ET , Schuster BG . First‐time‐in‐humans safety and pharmacokinetics of WR 238605, a new antimalarial. Am J Trop Med Hyg 1998; 58: 645–649. 9598455 10.4269/ajtmh.1998.58.645

[4] Llanos‐Cuentas A , Lacerda MV , Rueangweerayut R , et al. Tafenoquine plus chloroquine for the treatment and relapse prevention of *Plasmodium vivax* malaria (DETECTIVE): a multicentre, double‐blind, randomised, phase 2b dose‐selection study. Lancet 2014; 383: 1049–1058. 24360369 10.1016/S0140-6736(13)62568-4

[5] Chu CS , Freedman DO . Tafenoquine and G6PD: A Primer for Clinicians. J Travel Med 2019; 26: taz023. 30941413 10.1093/jtm/taz023 PMC6542331

[6] Shanks GD , Oloo AJ , Aleman GM , et al. A new primaquine analogue, tafenoquine (WR 238605), for prophylaxis against *Plasmodium falciparum* malaria. Clin Infect Dis 2001; 33: 1968–1974. 11700577 10.1086/324081

[7] Minucci A , Moradkhani K , Hwang MJ , et al. Glucose‐6‐phosphate dehydrogenase (G6PD) mutations database: review of the “old” and update of the new mutations. Blood Cells Mol Dis 2012; 48: 154–165. 22293322 10.1016/j.bcmd.2012.01.001

[8] Chu CS , Bancone G , Nosten F , et al. Primaquine‐induced haemolysis in females heterozygous for G6PD deficiency. Malar J 2018; 17: 101. 29499733 10.1186/s12936-018-2248-y PMC5833093

[9] Antibiotic Expert Group . Therapeutic Guidelines: Antibiotic. Version 16. Melbourne: Therapeutic Guidelines, 2019 https://www.tg.org.au/ (viewed Dec 2019).

[10] World Health Organization . Guidelines for the treatment of malaria; 3rd ed Geneva: World Health Organization, 2015 https://apps.who.int/iris/handle/10665/162441 (viewed Dec 2019).

[11] Bancone G , Gornsawun G , Chu CS , et al. Validation of the quantitative point‐of‐care CareStart biosensor for assessment of G6PD activity in venous blood. PLoS One 2018; 13: e0196716. 29738562 10.1371/journal.pone.0196716 PMC5940185

[12] Pal S , Bansil P , Bancone G , et al. Evaluation of a novel quantitative test for glucose‐6‐phosphate dehydrogenase deficiency: bringing quantitative testing for glucose‐6‐phosphate dehydrogenase deficiency closer to the patient. Am J Trop Med Hyg 2019; 100: 213–221. 30350771 10.4269/ajtmh.18-0612 PMC6335905

[13] Ley B , Bancone G , von Seidlein L , et al. Methods for the field evaluation of quantitative G6PD diagnostics: a review. Malar J 2017; 16: 361. 28893237 10.1186/s12936-017-2017-3 PMC5594530

[14] GlaxoSmithKline . Highlights of prescribing information: KRINTAFEL (tafenoquine). 2018 https://www.accessdata.fda.gov/drugsatfda_docs/label/2018/210795s000lbl.pdf (viewed Feb 2019).

[15] Lacerda MVG , Llanos‐Cuentas A , Krudsood S , et al. Single‐dose tafenoquine to prevent relapse of *Plasmodium vivax* malaria. N Engl J Med 2019; 380: 215–228. 30650322 10.1056/NEJMoa1710775 PMC6657226

[16] Llanos‐Cuentas A , Lacerda MVG , Hien TT , et al. Tafenoquine versus primaquine to prevent relapse of *Plasmodium vivax* malaria. N Engl J Med 2019; 380: 229–241. 30650326 10.1056/NEJMoa1802537 PMC6657225

